# Sponges: A Reservoir of Genes Implicated in Human Cancer

**DOI:** 10.3390/md16010020

**Published:** 2018-01-10

**Authors:** Helena Ćetković, Mirna Halasz, Maja Herak Bosnar

**Affiliations:** 1 Laboratory for Molecular Genetics, Division of Molecular Biology, Ruđer Bošković Institute, 10000 Zagreb, Croatia; mimesek@irb.hr; 2 Laboratory for Protein Dynamics, Division of Molecular Medicine, Ruđer Bošković Institute, 10000 Zagreb, Croatia; mherak@irb.hr

**Keywords:** porifera/sponge, evolution, cancer, cancer genes, molecular oncology

## Abstract

Recently, it was shown that the majority of genes linked to human diseases, such as cancer genes, evolved in two major evolutionary transitions—the emergence of unicellular organisms and the transition to multicellularity. Therefore, it has been widely accepted that the majority of disease-related genes has already been present in species distantly related to humans. An original way of studying human diseases relies on analyzing genes and proteins that cause a certain disease using model organisms that belong to the evolutionary level at which these genes have emerged. This kind of approach is supported by the simplicity of the genome/proteome, body plan, and physiology of such model organisms. It has been established for quite some time that sponges are an ideal model system for such studies, having a vast variety of genes known to be engaged in sophisticated processes and signalling pathways associated with higher animals. Sponges are considered to be the simplest multicellular animals and have changed little during evolution. Therefore, they provide an insight into the metazoan ancestor genome/proteome features. This review compiles current knowledge of cancer-related genes/proteins in marine sponges.

## 1. Introduction

While cancer evolution is still not fully understood, it is clear that the appearance of multicellularity is directly related to the origin of this widespread disease. Cancer can be described as a consequence of errors within the multicellular system causing the proliferation of “selfish” cell lines [1]. It is most likely that cancer appeared in parallel with multicellularity and the development of true tissues and organs, and that the genes responsible for cellular cooperation and multicellularity evolved together. When malfunctioning, these genes may cause cancer [2]. Intensive research in the field of molecular oncology over the recent decades expanded the initial division of cancer genes to oncogenes and tumor suppressor genes, by involving genes connected to angiogenesis, differentiation, adhesion, as well as to invasion processes, inflammation etc. Although little is known about cancer in invertebrates, especially non-bilaterians and unicellular relatives of animals, the available literature sources report on tumorous formations in some phyla, for example flatworms, molluscs, and insects [1]. Even some of the simplest “early branching” non-bilaterian animals, such as cnidarians, may develop tumors [3]. Comparative genomics studies have implied that a number of genes linked to human diseases were already present in species distantly related to humans [4] and similar has been suggested for cancer genes [5]. It seems that most of cancer-related protein domains have appeared during the two main evolutionary peaks, the emergence of unicellular eukaryotes and the transition to multicellular metazoans [5]. Since no systematic studies were thus far performed to reveal the occurrence of tumors in invertebrates, the current knowledge about cancer and status of cancer-related genes in simple animals is scarce. Studying cancer-related genes at the level at which they have first emerged, in a simpler model system, may provide an insight into their basic cellular functions.

The sponges (phylum Porifera) are the simplest and likely the most ancient group of animals that have separated from other metazoans more than 800 million years ago [6]. They are sessile as adults and lack true tissues and organs as well as any recognizable sensory or nerve structures. Despite their simple morphology, sponges genomes are complex [7] and many of their genes bear a striking similarity to vertebrate homologs [8]. Should we postulate that tumors had developed together with the emergence of tissues and organs, it would be likely for sponges to be unable to form tumors. Their genomes nevertheless, contain the majority of genes whose human homologs are implicated in cancer [7]. Research of cancer associated genes in simple animals with no tissues and blood vessels, such as sponges, could help in understanding the more complex signalling circuitry of their homologs in higher animals, such as vertebrates. Although comparative genomics studies have shown that many genes linked to cancer diseases in humans were already present in marine sponges [5,8], a precise understanding of their status and especially their biochemical and biological functions in sponges is more than limited.

**Figure 1:**
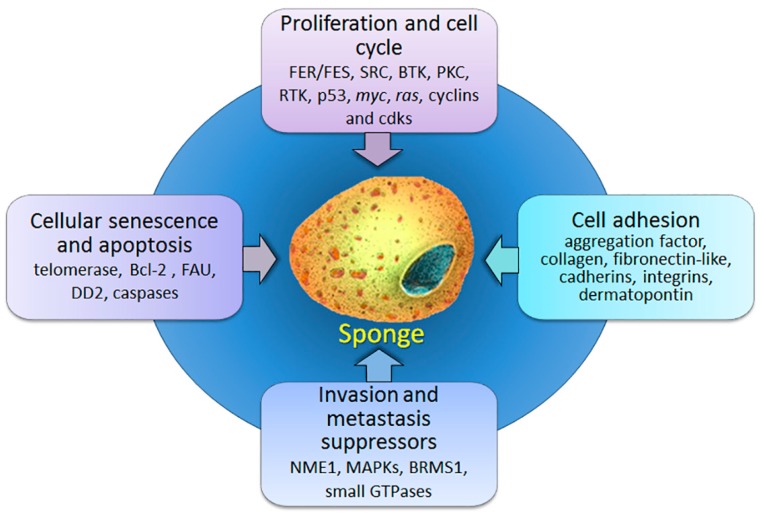
Schematic illustration of described sponge homologs of human cancer-related genes/proteins and cancer-associated processes.

Herein, we review the current knowledge on cancer-related genes/proteins in marine sponges (Figure 1) as well as the available experimental data about their biochemical characteristics and functions. Although genes/proteins are roughly divided according to their most prominent function, most of them are known to be involved in several cancer-causing processes.

## 2. Proliferation and Cell Cycle

The fundamental feature of cancer growth involves continuous, unsustained proliferation which eventually leads to tumor mass formation. Cell division in multicellular organisms is normally tightly controlled by numerous mechanisms which maintain a sophisticated balance between positive and negative regulators of cell proliferation. The controlled progression through the cell cycle ensures the well-being of an organism as a whole. Numerous cellular key players control these events, including receptors on the cell surface, various signalling molecules that conduct signals from the membrane to the nucleus, as well as transcription factors and repressors which enable or impede transcription of particular genes [9]. Numerous genes/proteins linked to proliferation and cell cycle progression have been described in sponges (Porifera). First and foremost are protein kinases, which, among various signalling pathways, also control cell proliferation [10].

### 2.1. Protein Kinases

The protein kinase family encompasses enzymes that catalyse transfer of the phosphate group from a high energy molecule such as adenosine triphosphate (ATP) to a specific substrate. Protein kinases play a significant role in cell-life regulation: in signal transmission pathways, cell differentiation control, proliferation, metabolism, DNA damage repair, cell motility, response to external stimuli, and apoptosis. Mutations, or other genetic alterations causing changes in their activity, often result in malignant transformation.

**Protein kinase C.** Protein kinase C (PKC) is a class of serine/threonine kinases which represents one of the most studied signaling kinases [11]. Although the expression of PKCs is changed in multiple cancer types, their diverse biological functions make it difficult to define the relationship between these changes and the initiation of a disease. Analysis of the gene coding for PKC in the sponge *Geodia cydonium* showed that it contains 13 exons and 12 introns and that exons encode the regulatory and catalytic domains typical for the metazoan PKCs. Further analysis of the promoter activity revealed that this, phylogenetically oldest, PKC gene contains a promoter functional in the heterologous mammalian cell system [12]. It was shown that sponge aggregation factor (AF) functions not only as a cell adhesion molecule, but also as a mitogenic agent [13]. In this context, PKC is involved in the AF induced transmembrane signalling. The activation of PKC leads to phosphorylation of many nuclear components, including the topoisomerase II, which subsequently activates the DNA replication process [14]. Two PKCs, *GCPKC1* and *GCPKC2*, have been sequenced from *G. cydonium* [15]. A comparison of the complete structures of the sponge PKCs, with those of higher metazoans, but also of protozoan, plant, and bacterial Ser/Thr kinases, revealed that the animal kinase domains display homologies with those from plants, protozoa, and bacteria. This implies that the Ser/Thr kinase domain has an universal common ancestor. However, the overall structure of the metazoan PKCs differs from non-metazoans, which suggests their distinct functions [15].

### 2.2. Protein Tyrosine Kinases

Protein tyrosine kinases (PTKs) specifically phosphorylate tyrosines on their target proteins. According to their cellular localization PTKs are divided in two major categories: receptor PTKs or transmembrane proteins (RTKs) and non-receptor or cytoplasmic PTKs. They are almost exclusively found in Metazoa and many have been described in sponges [16,17,18,19,20,21].

**The receptor tyrosine kinase.** The phylogenetic analysis of the protein-serine/threonine kinases (PS/TKs) from three sponges, the demosponges *G. cydonium* and *Suberites domuncula* and the calcareous sponge *Sycon raphanus*, suggests a common ancestry of PTKs with the PS/TK superfamily, from which the *G. cydonium* RTKs have diverged first [22]. The analysis of the *G. cydonium* RTK gene revealed that it contains introns outside of its TK domain, unlike the introns in higher animals that are inserted into the TK region [23]. Since the RTK from *G. cydonium* has been identified as the phylogenetically oldest member of PTKs [24], it was assumed that introns within the TK domains of genes from higher animals were inserted after the sponge taxa have branched off from all other metazoans [23].

**The FES/FER non-receptor tyrosine kinases.** Two different forms of Feline Sarcoma and FES Related proteins (FES/FER) exist in mammals [25] and both can be activated by a number of extracellular signals [26]. FER/FES non-RTKs are engaged in cytoskeletal rearrangements, as well as in cell-matrix and cell-cell interactions, while genetic analyses implicate their involvement in the regulation of inflammation and innate immunity [27]. The implication of FES and FER in human pathology still remains to be fully elucidated, but their high oncogenic potential has been implied in several recent studies [28,29]. The analysis of cDNA from the sponge *S. raphanus* disclosed a protein highly similar in its primary structure and organization of domains with tyrosine kinases (TKs) from the FES/FER family of non-RTKs [18]. The protein from *S. raphanus* was named FES/FER_SR since it exhibited high homology to the mammalian FES/FER proteins. Phylogenetic analysis revealed that FES/FER_SR from *S. raphanus* is the most ancient known member of the FES/FER family of non-RTKs [18]. Their role in organisms without tissues and organs, such as sponges, is not yet clear.

**The SRC non-receptor tyrosine kinases.** SRC (Rous sarcoma oncogene cellular homolog) is a non-receptor PTK that has been implicated in the development of malignant tumors in humans [30]. SRC is involved in many signaling pathways, such as gene transcription, cell cycle progression, cell adhesion, apoptosis, transformation, and migration. An extensive analysis of SRC PTKs from the marine sponge *S. domuncula* revealed nine different cDNAs encoding three different SRC proteins, SRC1SD, SRC2SD, and SRC3SD [16]. Considering that the N-termini of each of these proteins are unique, it can be speculated that the three sponge SRC kinases have different biological functions. Furthermore, it seems that all three SRCs from *S. domuncula* display the highest homology with SRCA PTKs from higher Metazoa. Phylogenetic analysis showed that SRC-related Src Homology 2 and 3 (SH2 and SH3) domains from different species are more conserved than SH2 and SH3 domains within different *S. domuncula* proteins [16]. The described data suggests that *src* genes are very ancient, that they already existed in the common ancestor of all Metazoa and that the ancestral *src* gene was already a multidomain protein composed of SH3, SH2 and tyrosine kinase (TK) domains.

**Bruton’s tyrosine kinase.** Bruton’s tyrosine kinase (BTK) belongs to the TEC (Tec Protein Tyrosine Kinase) family of PTKs and is required for maturation of the b-lineage lymphoid cells. Mutations in the BTK gene were shown to cause X-linked agammaglobulinemia (XLA) in humans and X-linked immunodeficiency (Xid) in mice [31,32]. Poriferan BTK-like protein (BTKSD) has been described from the marine sponge *S. domuncula*. This 700-aa-long protein contains all typical TEC family domains and shows the highest homology with the human BTK protein [17]. Phylogenetic analysis of PTKs from the TEC family revealed that the BTK/TEC genes have an ancient origin and have remained highly conserved during evolution [33]. These genes have probably been present in the common ancestor of all metazoans before gene duplications and the separation of sponges from other animal lineages. The structural analysis of the sponge and human promoter implies similar regulation of both genes; the kinase activities of the sponge BTKSD enzyme and its human homolog are in the same range [33]. Therefore, it is reasonable to presume that the ancestral-type protein was structurally and functionally similar to the multifunctional enzyme present in higher animals. Given that the BTK function in mammals is associates with the modulation of actin polymerization, a similar role of BTK-like protein in sponges is also expected. Modulation of actin polymerization by TEC family kinases probably has a significant role in cellular processes such as proliferation, motility, and adhesion [34]. Further, it was shown that PTKs of the TEC family possess a key role in production of the lipopolysaccharide (LPS)-induced tumor necrosis factor involved in the cellular response to bacterial infections [35]. Since *S. domuncula* is very efficient in fighting bacterial infections [36], it is possible that the role of BTK in sponges also includes antimicrobial defence.

**Cyclins, cyclin-dependent kinases, and other key proliferation molecules.** Cyclin-dependent kinases (CDKs) are a group of serine/threonine kinases, critical in the regulation of the cell cycle [37]. A comprehensive evolutionary study of cyclins and CDKs in metazoans and their unicellular relatives has been published by Lihuan Cao et al. [38]. It seems that the sponge genome lacks three cyclin subfamilies, cyclin A, cyclin D, and cyclin O, while the CDK4/6 subfamily is also missing from its genome. Although it has already been present in the placozoan *Trichoplax adhaerens*, the authors speculate that the establishment of the CDK4/6-cyclin D complex may have been fully functional in cell cycle control during eumetazoan emergence.

Finally, it seems that p53 (tumor protein p53), the guardian of the genome and putative tumor suppressor gene, is also present in the sponge genome as well as the powerful MYC (myelocytomatosis oncogene cellular homolog) and RAS (Rat sarcoma) oncogenes [7,39]. It has even been reported that the choanoflagellate *Monosiga brevicollis* already contains a subset of transcription factors, such as p53 and MYC, previously thought to be specific for metazoans [40]. The evolutionary history of p53 gene family as well as MYC [41] has been studied, but not specifically in the sponge genome [42]. The RAS-like family of proteins will be discussed further in the text.

## 3. Players in Cellular Senescence and Apoptosis

It is widely accepted that normal cells are able to pass only through a limited number of divisions. The unlimited proliferation of cancer cells has been associated with impairment of two major cellular cancer defence mechanisms: senescence and crisis/apoptosis. Senescence is normally a consequence of shortening of telomeres, multiple tandem hexanucleotide repeats that protect the ends of chromosomes from end-to-end fusion. The progressive shortening of telomeres can be stopped by telomerase, which can add repetitive nucleotide sequences to the ends of chromosomes [43]. In eukaryotic cells the telomere activity is usually restricted to germ and stem cells. In all other normal cells after a limited number of divisions the cell enters a senescent, but viable state. If the cell breaks this barrier, the crisis occurs which immediately redirects the cell to engage in the apoptotic program. The rare event of cellular immortalization of potential cancer cells occurs by the knock-down of both of these barriers. This is usually due to unlocking the transcriptional program which leads to dedifferentiation followed by upregulating of telomerase expression [9].

As expected, telomeres are an integral part of sponge chromosomes. The telomere sequence has been analyzed in three different sponge species and it consists exclusively of (TTAGGG) *n*-type sequences that are widely distributed in many multicellular animals belonging to the various phyla and classes [44]. The telomerase activity was determined in two different sponges: *S. domuncula* and *G. cydonium* [45]. A quantitative analysis revealed that tissues from both sponges contained telomerase activity of approximately 30% and 20%, respectively, of the activity in the positive reference cells. In higher animals the high telomerase activity is strictly limited to germ cells and metastatic tumors, while stem cells, needed for tissue renewal and injury repair, have intrinsically low levels of telomerase [46]. It is known that sponge tissue has a very low germ cell number, so this finding may indicate that all of the sponge cells have some, low telomerase level and, therefore, maintain permanently their proliferation potential. The isolated sponge cell in cell culture, however, stops proliferating [47] and loses the telomerase activity, opposed to the human tumor cells, which in culture conditions obtain both proliferation and telomerase activity. All these findings indicate that sponges have already developed the senescence/apoptotic program.

Apoptosis is a form of programmed cell death which can be found in multicellular metazoans and is a necessary prerequisite for the optimal functioning of these organisms. Apoptosis is a highly controlled mechanism which serves to eliminate redundant, infected, necrotic, or simply unwanted cells during development, senescence, morphogenesis, organogenesis, and defence, or other physiological and pathophysiological events [48]. In multicellular organisms apoptosis serves as a major barrier against uncontrolled proliferation, which leads to cancer. The apoptotic process can generally be divided into two pathways. The extrinsic pathway is triggered upon ligation of tumor necrosis factors (TNFs) transmitting the apoptotic signal into the cell. The intrinsic pathway is stress-dependent and controlled by members of the Bcl-2 protein family. Both pathways culminate with the activation of proteolytic enzymes, caspases, the executors of programmed cell death.

It seems that the sponge genome possesses components of both pathways. Experimental evidence proves that marine sponges, like *G. cydonium* or *S. domuncula*, undergo the process of apoptosis during formation of asexual reproduction bodies or in stress response [49]. The poriferan pro-apoptotic molecule DD2 (death domain 2), a death domain containing molecule, was identified by screening *G. cydonium* library with degenerate primers directed towards a conserved region of human FAS receptor (fas cell surface death receptor). Interestingly, this protein has two death domains which have not been found in any known protein involved in the TNF signalling pathway.

Moreover, evolutionary ancient members of the intrinsic, Bcl-2 (B-cell lymphoma 2) family members have been identified in sponge [50]. The sponge *G. cydonium* expresses a pro-survival Bcl-2 homolog BHP2 that impairs apoptosis in sponge tissue induced by heat shock or tributyltin exposure [51]. Recently, Caria et al. [52] performed structural and binding studies on BHP2 and BAK-2 (Bcl-2 Antagonist/Killer) from *G. cydonium* and the freshwater sponge *Lubomirskia baicalensis*. The results of their findings suggest that the molecular machinery and mechanisms for executing Bcl-2-mediated apoptosis are evolutionary ancient and resemble their mammalian versions.

Furthermore, FAU (Finkel-Biskis-Reilly murine sarcoma virus (FBR-MuSV) ubiquitously expressed), recently identified as a pro-apoptotic regulatory gene mediated by Bcl-G (a pro-apoptotic member of the Bcl-2 family), has also been identified in the sponge genome [53]. FAU gene encodes a fusion protein composed of an ubiquitin-like protein (FUBI) at its N terminus and ribosomal protein S30 at the C terminus. The protein is then post-translationally processed into two separate proteins, FUBI and S30 ribosomal protein. *FAU* has been identified as a pro-apoptotic regulatory gene, whose expression has been downregulated in human ovarian, prostate, and breast cancer [54,55,56]. A study of the FAU protein from the marine sponge *S. domuncula* showed that sponge FAU protein displays a pro-apoptotic activity, as it increases apoptosis in human HEK293T cells (human embryonic kidney cells) as well as its human homolog and was shown to be more similar to its human homolog than to the one from *Caenorhabditis elegans* or *Drosophila melanogaster*. These findings implicate that the common metazoan ancestor probably possessed the FAU protein that was structurally and functionally similar to its recent version and has changed very little during evolution [57]. Additionally, the number, phases, and position of its introns were not significantly changed from sponges to humans. Ribosomal proteins (RPs) are evolutionary conserved components of ribosomes crucial for the correct ribosome assembly. Many ribosomal protein genes (RPGs) have additional extraribosomal functions, being involved in replication, regulation of cell growth, apoptosis, and cancer development [58]. RPs have shown to be important tools for studying intron evolution, since the analysis of RPG introns in sponges revealed the same rate of conservancy between two different sponge species compared to the conservancy between sponges and humans [59,60]. Since S30 ribosomal protein is produced from the fusion protein transcribed from the pro-apoptotic regulatory gene FAU, its extraribosomal function may be connected to the regulation of apoptosis [57].

As mentioned above, the apoptotic process is eventually executed by specific proteases, caspases. According to Wiens et al. [49], altogether three caspase-like proteins have been found in Porifera. The smaller number of caspase family genes, compared to vertebrates, probably implies the simplicity of this proteolytic cascade in sponges, but undoubtedly suggests that this process was already present and engaged in first simple multicellular animals.

## 4. Cell Anchoring Molecules

Adhesion of cells is a primary prerequisite for tissues architecture. Cell within tissues adhere to each other, as well as to the extracellular matrix (ECM); these connections are collectively called the anchoring junctions. Several types of anchoring junctions have been identified, depending on the type of adhesion, and they are in general responsible not only for anchoring cells within a certain tissue, but also for their migration. Anchoring junctions are multiprotein complexes in which cell-adhesion molecules (CAMs) play a crucial role. There are five principal classes of CAMs: cadherins, immunoglobulin (Ig) superfamily, selectins, mucins, and integrins. Integrins mediate cell-matrix interactions whereas other types of CAMs participate in cell–cell adhesion [61]. Cell adhesion is essential in all aspects of vertebrate cell growth, migration, and differentiation. CAMs are involved in a wide variety of cellular functions including signal transduction, cellular communication and recognition, embryogenesis, inflammatory and immune responses, and apoptosis [62], therefore, they play a significant role in cancer progression and metastasis. For example, tumor invasion and metastasis frequently coincide with the loss of E-cadherin-mediated cell–cell adhesion [63].

During the last century sponges have been repeatedly used as a model system for studying cell adhesion [26,64]. The first extracellular particle named aggregation factor (AF), which promotes the species-specific aggregation of sponge cells, was isolated from two sponges in 1973 [65,66]. The *G. cydonium* AF was shown to bind the plasma membrane-bound aggregation receptor (AR), after which the RAS gene is expressed [67]. The RAS gene product further binds to the anti-aggregation receptor (aAR), which has been identified as a lectin receptor [68,69]. After the first cell–cell adhesion molecule, galectin [70], and the first cell–matrix adhesion receptor, integrin [71,72], were identified in sponge *G. cydonium*, it became clear that sponges contain proteins involved in adhesion, which also show homology to those in humans [73]. The extracellular matrix (ECM) represents a platform for cell adhesion via integrin receptors, signal transduction, cell development, and cell division. Sponge mesohyl, a space between the external pinacoderm and the internal choanoderm, is composed of collagen, fibronectin-like molecules, and dermatopontin [74], which are also found in the ECM of higher metazoans. Therefore, it seems that the sponge mesohyl could serve as a primitive ECM in sponges.

**Cadherins.** Cadherins (CDHs) are a major component of adherent junctions, typically present in vertebrate epithelial tissues. Adherent junctions are composed primarily of type I cadherins, transmembrane glycoproteins that form Ca^+2^-dependent homotypic complexes. Loss of cadherins enables cancer cells to detach from the original tissue and trigger the metastatic cascade. It is widely accepted that the key molecule in metastasis onset is E-cadherin, specifically. Cadherins (CDHs) and cadherin-related proteins are found in the genomes of all sequenced metazoans, including diverse bilaterians, cnidarians, sponges, and also in choanoflagellates, the closest unicellular relatives of animals [75]. CDH1 and CDH2 (type I) are found to be involved in metastasis suppression. Nichols et al. [76] have described the incomplete type I CDH1 from the draft genome of sponge *Oscarella carmela*.

**Integrins.** Integrins form a large family of cell adhesion receptors involved in cell adhesion, migration, and signalling [77]. Integrins are heterodimers composed of one α and one β subunit. Eighteen different integrin α subunits and eight β subunits have been described [78].

All animals express integrins, indicating that this family evolved early in the history of metazoans. Both α and β subunits have been found throughout the invertebrates species, including sponges [71,72,79].

## 5. Metastasis Suppressor Genes

Metastasis is a process in which tumor cells detach from the original tissue, travel to nearby or distant sites in the body and form secondary tumors. Metastasis suppressors inhibit the tumor cell ability to metastasize, having little or no effect on the primary tumor growth. Metastasis suppressors are involved in one or several steps of the metastatic cascade, which includes the following: detachment of the cell from the main tissue, breaking through the basal lamina and invasion of the surrounding tissue, entrance to the nearby blood or lymphatic vessels, surviving the transit through the lymphatic or blood system and extravasation from blood/lymphatic vessels into the distant tissue, where they can grow into macroscopic tumors [80]. The ongoing discoveries of genes/proteins that are directly involved in the metastatic cascade seem to be an important step forward in our understanding of this process.

**NME1.** The human NME1 (non-metastatic 1) is the first identified and the most studied metastasis suppressor [81], present in all three domains of life: Bacteria, Archaea, and Eukarya [82]. The NME1 protein possesses the nucleoside diphosphate kinase (NDPK) activity, but also has some additional functions such as histidine kinase activity, transcription factor activity, etc. [83,84]. A single gene coding for the human NME1 homolog was found in the genome of the marine sponge *Amphimedon queenslandica* [7] and *S. domuncula* cDNAs [8,85]. These sponge homologs are similar in primary structure and possess conserved residues essential for NDPK activity. Biochemical and functional characterizations of non-bilaterian homologs of the human NME1 protein are still scarce. Our group has shown that sponge genes NMEGp1 are intron-rich and that these introns are relatively short. The analysis of sponge NMEGp1 promoters showed that some of the motifs crucial for human promoter activity are also present in sponges. Furthermore, the analysis of protein activity showed that the sponge *S. domuncula* NMEGp1Sd protein possesses a hexameric form and has a similar level of kinase activity as the human NME1 protein. The sponge homolog interacts with its human ortholog/homolog in human cultured cells and shows the same subcellular localization pattern as human NME1. Also, if expressed in human tumor cells, the sponge homolog significantly reduces its migration potential. Based on the evidence presented, we have concluded that the sponge NME protein can replace its human homolog in at least some of its biological functions which are usually associated with “higher” metazoans. We also presumed that the biochemical function of NME1, which is responsible for metastasis suppression in human, is present in the sponge, and was possibly also present in the ancestor of all Metazoa [86,87]. On the contrary, our studies on another member of the NME family in sponges, NME6, indicates that this ancient gene/protein probably changed its biochemical/biological function during the course of evolution. It is not yet elucidated whether this protein is also linked to tumor formation or progression [88].

**Small GTPases.** RAS-like superfamily of GTPases (small GTPases) are a family of enzymes that bind and/or hydrolyze GTP. The state of the bound nucleotide acts as a switch between the active and inactive state of the enzyme. The RAS superfamily is divided into five subfamilies: RAS, RHO, RAN (Ras-related nuclear protein), RAB (Ras genes from rat brain), and ARF (ADP ribosylation Factor) [89]. Small GTPases play an important part in numerous cellular processes but it is generally accepted that the RAS family is responsible for cell proliferation, RHO for cell morphology, RAN for nuclear transport, and RAB and ARF families for vesicle transport. Therefore, it is clear that the RAS-like superfamily is implicated in several aspects of cancer occurrence and progression [90]. The analysis of the EST database from the marine sponge *S. domuncula* has revealed cDNA sequences encoding 50 different RAS-like small GTPases. Forty-four sponge proteins from the RAS family are described here: six proteins from the RAS subfamily, five from RHO, six from ARF, one RAN, and 26 RABs or RAB-like proteins. Small GTPases from sponge are more related in their primary structure to the orthologues from vertebrates than to those from other invertebrates. These findings imply that diversification of genes encoding RAS-like small GTPases happened after the duplications that occurred very early in the evolution of Metazoa [39].

## 6. Cancer Associated Genes in Marine Sponges-Final Remarks and Future Challenges

Apart from humans, tumors have mostly been described in other vertebrates, especially in farm animals and pets, as well as in other animals kept in captivity [1]. Furthermore, they have also been reported in invertebrate deuterostomes [1], protostomes [80,91], and even in simple non-bilaterian animals [3,92,93], although these findings should be taken with caution since laboratory breeding and culture conditions may be far from those found in their natural habitats [3]. Therefore, it is doubtful whether these organisms would develop tumors also in a natural environment. Indirect evidence of the presence of tumors in simple non-bilaterian animals also comes from the fact that marine invertebrates, especially sponges, produce bioactive compounds [94], some of which have an antitumor activity on human cells in culture [95,96,97]. The role of these compounds in sponge biology is not fully understood. Since, sponges are sessile and lack capabilities for physical defences, they are often targets of marine predators. Therefore, it is expected that sponges have developed a range of defensive chemicals in order to disable and repel predators. They also use their defensive substances to keep plants and other animals’ offsprings from settling onto their surface [98,99]. Although it is widely accepted that sponges use these compounds for ecological purposes it cannot be excluded that at least some of these substances, are used in physiological processes, e.g., as a primitive form of immunological defence, in the regulation of cell count or for various metabolic purposes. Some of the compounds they produce are directly engaged in specific signalling pathways. However, it should be taken into consideration that more than one pathway is usually affected by each of those compounds.

About a hundred novel compounds that exhibit significant inhibitory activity towards a range of protein kinases have been detected so far [100]. Some of these act as inhibitors of protein kinase C (PKC): xestocyclamine A [101] (*Z*)-Axinohydantoin), debromo-*Z*-axinohydantoin, hymenialdisine, debromohymenialdisine [102], frondosins A–E [103], BRS1 (C30 BIS-Amino, BIS-Hydroxy polyunsaturated lipid) [104], sesquiterpenoid quinones, nakijiquinones A–D [105,106], cytotoxic sesterpenes spongianolides A–E [107], lasonolide A [108,109,110], azetidine compound penazetidine A [111], and numerous others. Several compounds that display inhibitory activity towards CDKs have also been isolated from marine sponges, such as hymenialdisine [112,113], microxine, variolin B [114,115], fascaplysin [116], and konbu’acidin A [117]. It has also been shown that marine sponges possess tyrosine protein kinase (TPK) inhibitors: the penta-, hexa- and hepta-prenylhydroquinone 4-sulfates [118], melemeleone [119], and polyketide inhibitors [120]. Furthermore, (+)-aeroplysinin-1 from sponge displays a strong antitumor effect by blocking the proliferation of EGFR-dependent human breast cancer cell lines MCF-7 and ZR-75-1 [121], while curcuphenol displays a SRC protein kinase inhibition and curcudiola focal adhesion kinase (FAK) inhibition [122].

So far, as much as 60 compounds isolated from marine sponges have been shown to induce anticancer activity through apoptosis. For the majority of them, the proapoptotic mechanism has not yet been fully elucidated [123]. The pre-clinical study on Renieramycin M reveals that this substance induces apoptosis in cancer cell lines through the p53-dependent pathway and consequently inhibits cancer progression and metastasis [96]. Renieramycin M, a tetrahydroisoquinoline, is the first anticancer drug to be approved by the European Union and by the Food and Drug Administration (FDA) for the treatment of advanced soft tissue sarcomas [124]. Selected compounds for which cellular and molecular mechanisms were explored in depth include: aaptamines [125], psammaplysene A (PsA) [126], stellettin B [127,128,129], candidaspongiolide [130], heteronemin [131,132,133], and dideoxypetrosynol A [134]. Many compounds produced by marine sponges display antiproliferative activity targeting various signaling pathways of the cell-cycle progression such as aragusterol A [135], (19Z)-hlichondramide [136], smenospongine [137], and crambescidin 800 [138]. Some can also act as anti-inflammatory molecules targeting TLR (Toll-like receptor) signalling pathways [139], or as inhibitors of matrix proteinases [140]. Further, it has been shown that macrolide halichondramide (HCA) has an antimetastatic effect on human prostate cancer cells PC3, inhibiting their migration and invasion through the modulation of the crucial cadherin switches [141]. The specific role of these substances in sponge ecology and metabolism will hopefully be disclosed by future studies.

Based on the data collected so far, we can only speculate on the presence of cancer during the early animal evolution. It is presumed that tumors in invertebrates do not exhibit a malignant phenotype, while the progress in malignancy probably runs in parallel with the development of the immune system [142], as well as the development of a highly effective vasculatory system in vertebrates [143].

Despite the lack of true organs and tissues in sponges, they obviously possess a number of genes/proteins related to tumor onset and progression. Their genomes contain many gene homologs of those involved in human cancer development. Furthermore, the majority of sponge proteins transcribed from these genes possess the same domain organization as their homologs from higher metazoans, yet, their function within the sponge cells remains to be elucidated. Tumors in sponges have not been identified so far and, because of its simple structure, the formation of tumors within the sponge body is highly unlikely. Nevertheless, a number of studies indicated that sponge proteins may reflect at least some of the biochemical characteristics related to their homologs in higher animals, particularly humans. We know today that several other physiological processes are linked to tumor emergence, such as angiogenesis, inflammation, or cellular energetics [9]. Evidence shows that at least some of the components involved in these processes are present in sponges, but no systematic research is done in this context [7,144].

Sponges represent an important model, not only for studying ancestral metazoan homologs and their features before the diversification and specialization of these genes in higher animals, but also for understanding the basic physiological function of cancer genes in simpler animals. These findings could help to elucidate the more complex interactions of their homologs in humans and consequently explain possible reasons for their oncogenic potential.
